# The Cbk1-Ace2 axis guides *Candida albicans* from yeast to hyphae and back again

**DOI:** 10.1007/s00294-020-01152-1

**Published:** 2021-01-12

**Authors:** Rohan S. Wakade, Damian J. Krysan

**Affiliations:** 1 Department of Pediatrics, Carver College of Medicine, University of Iowa, Iowa City, IA 52242 USA; 2 Department of Microbiology/Immunology, Carver College of Medicine, University of Iowa, Iowa City, IA 52242 USA; 3 Departments of Pediatrics and Microbiology/Immunology, University of Iowa, ML 2040E, 25 South Grand Ave, Iowa City, IA 52242 USA

**Keywords:** *Candida albicans*, RAM network, Lateral yeast, Hyphae, Mitotic exit, G1

## Abstract

Since its description in *S. cerevisiae*, the Regulation of Ace2 and Morphogenesis (RAM) pathway has been studied for nearly 20 years in multiple model and pathogenic fungi. In pathogenic fungi, the RAM pathway carries out many functions through mechanisms that remain to be defined in detail. Recently, we reported that Cbk1-mediated phosphorylation of the transcription factor Ace2 functions to repress the hyphae-to-yeast transition in *Candida albicans*. This transition is understudied relative to the yeast-to-hyphae transition. Subapical hyphal cell compartments are arrested in G1 until the point at which lateral yeast emerge. Here, we discuss this model and report new data indicating that a second G1 associated protein, the mitotic exit regulator Amn1. In *S. cerevisiae* diploid cells, Amn1 negatively regulates Ace2 at both the gene expression level through a negative feedback loop and at the protein level by targeting Ace2 for degradation. In *C. albicans*, Amn1 and Ace2 also form a feedback loop at the level of gene expression. Deletion of *AMN1* decreases lateral yeast formation relative to wild type in maturing hyphae and is associated with decreased expression of *PES1*, a positive regulator of lateral yeast formation. These data indicate that the regulation of mitotic exit plays a role in determining the timing of lateral yeast emergence from hyphae in *C. albicans*. We also propose an integrated model for the interplay between the Cbk1-Ace2 axis and other hyphal stage regulators during the process of filamentation and transition back to yeast.

## Introduction

**Fig. 1:**
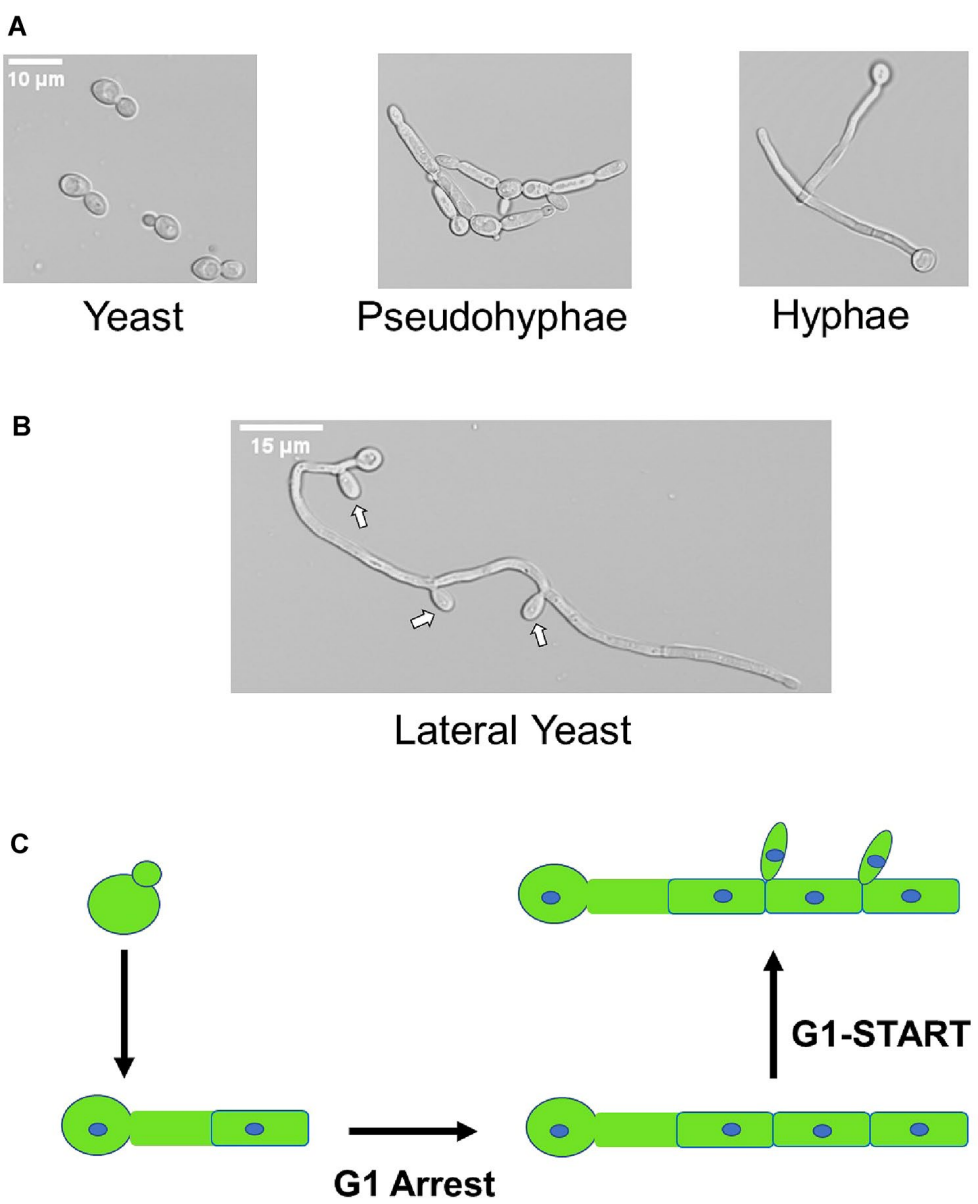
*Candida albicans* forms multiple morphological cells types. **a** Examples of yeast, pseudohyphal, and hyphal *C. albicans* cells. **b** Lateral yeast formation in mature hyphal cells. **c** Schematic indicating cell cycle state of sub-apical cells and the transition to lateral yeast formation

*Candida albicans* is an important human fungal pathogen that causes both superficial, mucosal infections and deep organ, invasive disease in both immunocompetent and immunocompromised individuals (McCarty and Pappas 2016). *C. albicans* is also part of the normal human mycobiome with primary niches in the gastrointestinal and genitourinary tracts (Romo and Kumamoto 2020). During infection, *C. albicans* adopts at least three distinct morphological cell types: the familiar budding yeast form; pseudohyphae; and true hyphae (Fig. 1a, for detailed definitions of each morphotype see: Sudbery 2011). Because the ability to transition between these morphotypes affects the virulence of *C. albicans*, the processes that regulate these transitions have been the subject of intensive study for decades (Arkowitz and Bassilana 2020). The vast majority of these studies have focused on the transition from yeast to hyphae or pseudohyphae because this transition is most tightly correlated with virulence in mammals.

A significantly understudied transition occurs after a hyphal cell has accumulated multiple sub-apical cell compartments. At this stage, yeast cells re-emerge from the hyphae and thus a hyphae-to-yeast transition occurs (Shen et al. 2008). These sub-apical cells are typically referred to as lateral yeast cells (Fig. 1b). Increased interest in the hyphae to yeast transition has been driven in part by the relatively recent recognition that it plays an important role in biofilm pathobiology (Uppuluri et al. 2010). Specifically, the outer layers of *C. albicans* biofilms release yeast cells or dispersed cells as the structure matures. Dispersed cells are thought to mediate dissemination of *C. albicans* from biofilms to other parts of the body and, thus, play an important role in the infection process (Wall et al. 2019).

Lateral yeast formation is delayed until several rounds of hyphal cell extension have occurred (Fig. 1c). The mother cells and sub-apical compartments are arrested in G1 while the apical compartment is mitotically active (Sudbery 2011). Gow and co-workers developed a model to explain the G1 arrest that is based on the observation that newly formed hyphal compartments are highly vacuolated and thus have relatively reduced cytoplasmic volume (Gow and Gooday 1987). This low cytoplasm content means that the hyphal compartments are equivalent to much smaller cells—i.e., new, small daughter cells. Cell size is a well-characterized determinant of cell cycle re-entry in yeast (Chaillot et al. 2017). Once the daughter cell has grown to a specific size it completes its transition from G1 to START. As the sub-apical compartments mature, the cytoplasm/vacuole ratio increases to the point where the cells exit from G1 and enter START (Barelle et al. 2003), leading to the emergence of lateral yeast cells from subapical compartments. Although this cell biological model nicely fits the observed data, almost nothing is known about the molecular details underlying the mechanism of the hyphae-to-yeast transition.

An important exception to this general statement comes from Kohler and co-workers who found that expression of the pescadillo homologue *PES1* is both necessary and sufficient to drive lateral yeast formation (Shen et al. 2008). In *C. albicans*, *PES1* is essential in yeast cells but dispensable in hyphae. Overexpression of *PES1* increases the formation of lateral yeast while decreased expression using a doxycycline-regulated allele reduced lateral yeast formation. In addition, *PES1* has been shown to play a role in the formation of biofilm dispersal cells.

How Pes1 regulates the hyphae to yeast transition remains unknown. In a second study, Lindsay et al. reported that farnesol induces lateral yeast formation (Lindsay et al. 2012). Farnesol inhibits hyphal morphogenesis by decreasing the activity of the Ras1-cAMP pathway. These data suggest that the emergence of lateral yeast is dependent on a down-regulation of the Ras1-cAMP pathway (Lindsay et al. 2012). Although these studies provide important initial clues to the regulation of the hyphae to yeast transition, much more remains to be learned. Here, we discuss our recent work on the role of the Regulation of Ace2 and Morphogenesis (RAM) pathway in the regulation of hyphal-related processes (Wakade et al. 2020) and present data providing new details regarding the role of the mitotic exit regulator Amn1 in the modulation of *ACE2* expression and the formation of lateral yeast cells from hyphae.

## Results and discussion

**Fig. 2:**
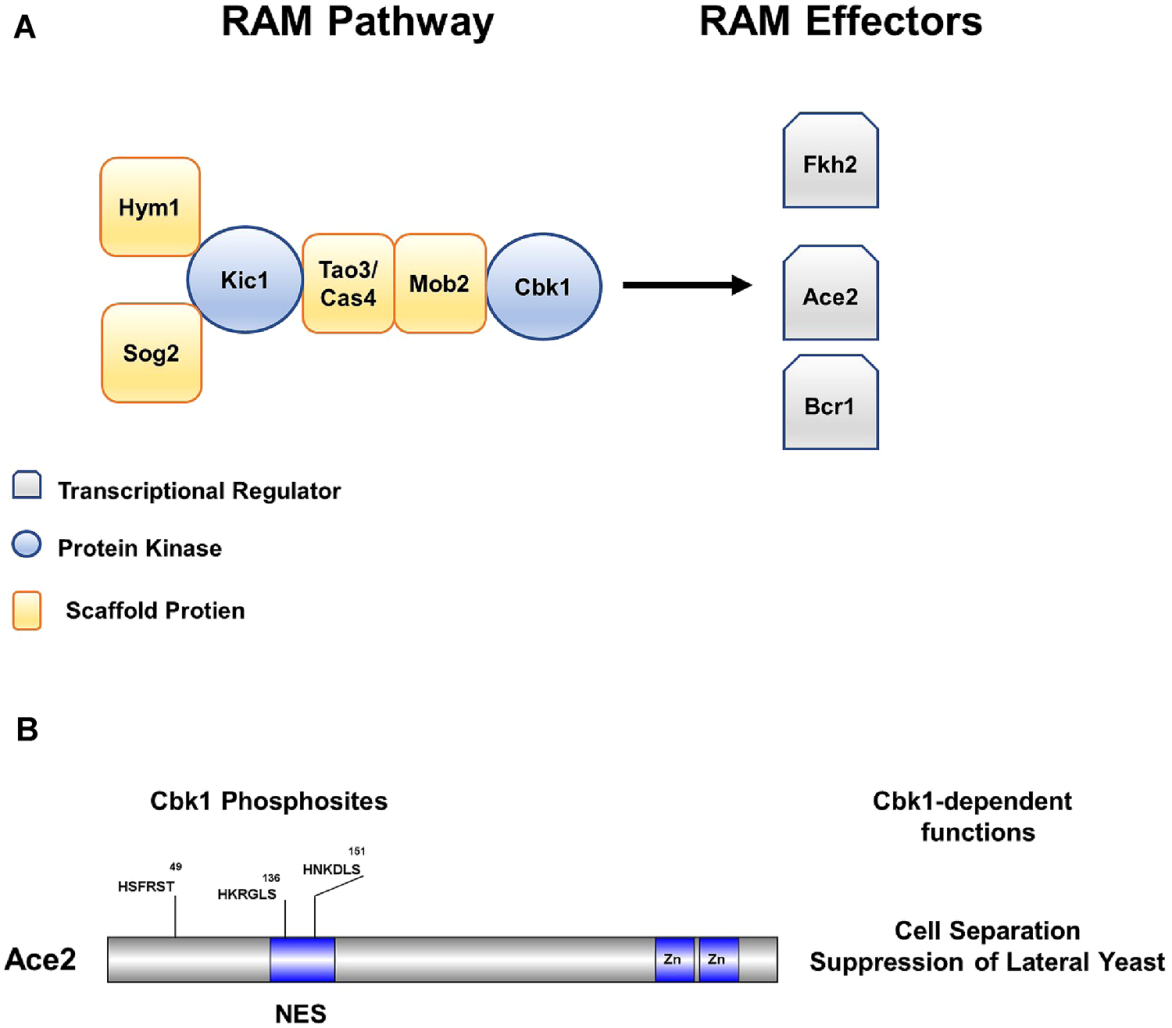
Regulation of Ace2 and Morphogenesis (RAM) pathway and Cbk1 phosphosites of Ace2. **a** Schematic showing the different components of the RAM pathway and examples of three Cbk1-regulated transcription factors in *C. albicans*. **b** The location of Cbk1 consensus phosphorylation sites and the Cbk1 dependent functions that were identified by mutation of those sites to non-phosphorylatable alanine (Wakade et al. 2020)

The RAM pathway (Fig. 2a) was initially described in *S. cerevisiae* and is conserved across both pathogenic and model fungi (Nelson et al. 2003; Saputo et al. 2012). The RAM pathway is made up of a Ndr/Lats kinase (Cbk1 in *C. albicans* and *S. cerevisiae*) along with an additional kinase Kic1 as well as four binding partners (Hym1, Sog2, Tao3/Pag1, and Mob2). The latter five proteins are required to activate Cbk1 with Mob2 directly binding Cbk1. The transcription factor Ace2 was incorporated into the pathway’s name because it was the first effector molecule shown to be dependent on the RAM pathway (Nelson et al. 2003). The most extensively studied function of the Cbk1-Ace2 axis is its role in daughter cell specific expression of septum-degrading genes required for cell separation (Sbia et al. 2008). Cbk1 phosphorylates Ace2 near its nuclear export signal during G1, trapping it in the nucleus (Mazanka et al. 2008). The Weiss lab established that Ace2 mutants lacking Cbk1 phospho-acceptor residues in *S. cerevisiae* display cell separation defects and have reduced expression of cell septum degrading enzymes such as chitinases and glucanases. Recently, we generated the analogous Cbk1 phosphosite mutants of Ace2 in *C. albicans* and showed that, as in *S. cerevisiae*, Cbk1 phosphorylation of Ace2 is required for the accumulation of Ace2 in the nuclei of daughter cells in yeast phase growth, expression of chitinases and glucanases, and ultimately, normal mother-daughter cell separation (Wakade et al. 2020).

*C. albicans* Cbk1 and, under specific conditions, Ace2 are also involved in filamentous growth. Cbk1 is absolutely required for filamentous growth due, at least in part, to its role in the negative regulation of Nrg1, a key repressor of the hyphal program (Lee et al. 2015). The best characterized role for Ace2 during filamentation is under hypoxic and embedded conditions where it is required for normal hyphal morphogenesis (Mulhern et al. 2006; Desai et al. 2016). We found that Cbk1 phosphorylation of Ace2 plays a modest role in filamentation under hypoxic conditions but no role when cells are embedded within agar (Wakade et al. 2020). Deletion of *ACE2* has a minimal effect on in vitro filamentation using standard liquid induction conditions such as serum (Saputo et al. 2014), Spider medium or other medium and we confirmed that was the case for strains lacking Cbk1 phosphorylation sites (Wakade et al. 2020).

During these experiments (Wakade et al. 2020), we noticed that *ace2*∆∆ strains developed lateral yeast cells at time points when the WT comparator had very few such cells. Specifically, after 4 h of induction in Spider medium at 37 °C, only 10% of hyphae formed by the SN250 reference strain displayed lateral yeast cells while 40% of hyphae formed by the congenic *ace2*∆∆ strain had formed lateral yeast cells. Mutation of the Cbk1 phosphorylation sites to alanine increases lateral yeast formation to levels (50%) that closely match the *ace2*∆∆ null mutant, indicating that Cbk1 phosphorylation of Ace2 leads to suppression of lateral yeast cell formation during hyphal morphogenesis (Wakade et al. 2020).

These results have novel implications for the function of Cbk1-Ace2 and for the regulation of lateral yeast formation. First, this is only the second regulatory pathway linked to the timing of lateral yeast formation (Lindsay et al. 2012). Second, Ace2 must have functions outside of the new daughter/apical cell during hyphal morphogenesis and thus functions in the subapical compartments of the hyphae. We and others have shown that Ace2 localizes to the apical cell nuclei of *C. albicans* hyphae but it is not detectable in either the cytoplasm or nuclei of sub-apical cells (Kelly et al. 2004; Bharucha et al. 2011). Paradoxically, despite being predominantly localized to the apical cell nuclei, Ace2 has no effect on the emergence of the new hyphal cell body while it has a significant effect on the biology of sub-apical cells in which it appears to be present at vanishingly low levels.

As mentioned above, *PES1* is necessary, and sufficient, for lateral yeast formation (Shen et al. 2008) and, consistent with this role, its expression is upregulated in *ace2*∆∆ deletion mutants relative to wild type hyphal cells (Wakade et al. 2020). At this point, it is not clear whether Ace2 directly suppresses *PES1* transcription or not. Our RNA sequencing data shows that a large set of genes (144) is upregulated in hyphal *ace2*∆∆ cells compared to wild type (Wakade et al. 2020); however, there are no biochemical data indicating that Ace2 is a direct repressor of gene transcription nor have chromatin immunoprecipitation experiments indicated that Ace2 binds to the promoter of *PES1* (Desai et al. 2016). Still, many transcription factors function as both activators and repressors.

Regardless of the specific mechanism by which loss Ace2 function triggers lateral yeast formation, the early development of lateral yeast does not affect the ongoing hyphae formation. The hyphae formed by *ace2*∆∆ are otherwise unchanged (Mulhern et al. 2006; Saputo et al. 2014). Furthermore, hypha-specific genes such as *HWP1* are expressed at normal or slightly increased levels in *ace2*∆∆ cells (Wakade et al. 2020). Consequently, both hyphal and yeast phase growth programs are operative over the length of the hyphae. As such, although Cbk1-activated Ace2 prevents the premature resumption of yeast cell morphogenesis, its role is distinct from factors that function in hyphal maintenance (Lu et al. 2014) because the hyphae program continues in its absence.

The function of Ace2 in the subapical cells is consistent with its cell cycle role as a G1 factor in *S. cerevisiae* (Sbia et al. 2008; Mazaka and Weiss 2010). Indeed, Ace2 has been proposed to delay G1 in daughter cells based on overexpression studies (Laabs et al. 2003). However, others have shown that the role of Ace2 may be more complex because deletion of *ACE2* appears to equalize the growth rate of mother and daughter cells rather than accelerate the cell cycle (Bogomolnaya et al. 2006). As mentioned earlier, the subapical cells of *C. albicans* are thought to be arrested in G1 (Gow and Gooday 1987; Sudbery 2011) and hence it seems likely that active Ace2 would be present in those cell compartments. Recently, the *S. cerevisiae* mitotic exit regulator Amn1 was reported to bind Ace2 and target it for ubiquitin-dependent degradation (Fang et al. 2018). Interestingly, Ace2 is also required for expression of *AMN1*, but only in *S. cerevisiae* diploid cells and not haploids. We were, therefore, interested to see if Amn1 played a role in the regulation of *ACE2* expression or lateral yeast formation.

**Fig. 3:**
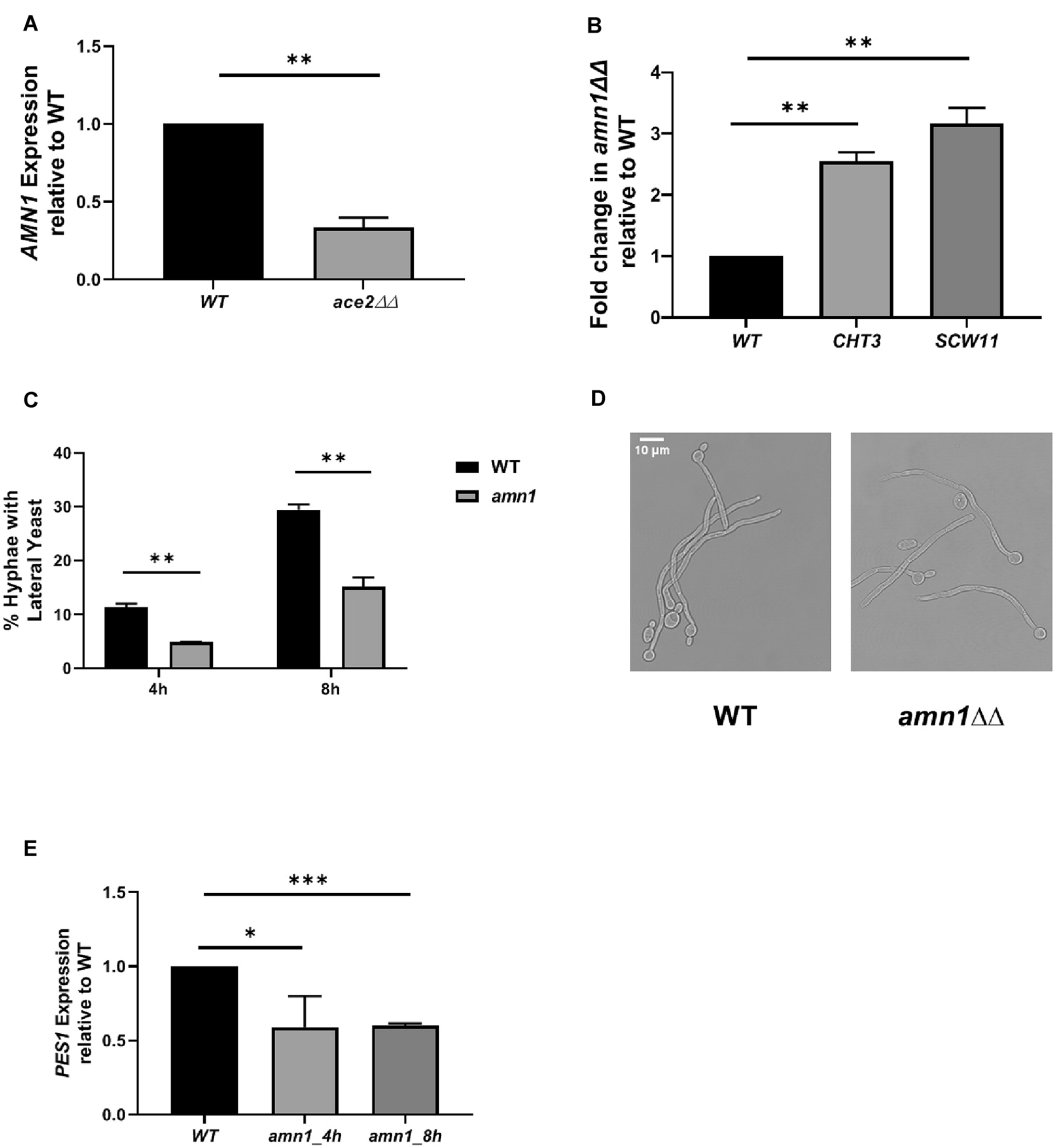
Transcriptional interplay between Ace2 and the G1 regulator Amn1 in *C. albicans*. **a** *AMN1* expression is decreased in *ace2*∆∆ in both yeast and hyphal phase as determined by RNA-sequencing experiments (Wakade et al. 2020). The effect was confirmed for hyphae using RT-PCR. **b** Deletion of *AMN1* increases the expression of Ace2 targets *CHT3* and *SCW11* in hyphae. **c** Lateral yeast emergence is delayed in *amn1*∆∆ mutants relative to wild type following hyphal induction in Spider medium at 37 °C. **d** Micrographs of WT and *amn1*∆∆ strains induced in Spider medium as for (**c**) showing that the deletion mutant forms true hyphae. **e** The expression of *PES1* was determined by RT-PCR 4 and 8 h after hyphal induction with Spider medium at 37 °C. For **a**, **b** and **e** bars indicate the mean ratio of the expression of the indicated gene expressed as fold-change in the ratio of mutant/wild type. Error bars indicate the standard deviation of two biological replicates performed in duplicate. Significant differences were determined by Student’s *t* test with a threshold of *P* < 0.05: *P < 0.05; **P < 0.01; ***P < 0.001. Bars indicate the number of hyphae with lateral yeast as determined by light microscopy. For **c**, at least 100 cells were characterized per replicate. The error bars indicate the standard deviation for at least two independent experiments. Student’s *t* test analysis of the difference between the wild type and *amn1*∆∆ cells indicated it was statistically significant (*P* < 0.05)

To explore the relationship between Ace2 and Amn1 further, we examined the expression of *AMN1* in *ace2*∆∆ during hyphae formation in our previously reported RNA seq data (Wakade et al. 2020). Indeed, *AMN1* expression is reduced in *ace2*∆∆ yeast cells hyphae relative to WT hyphae and we confirmed this in hyphal cells by RT-PCR (Fig. 3a). If Amn1 negatively regulates Ace2 function in *C. albicans*, then deletion of *AMN1* should lead to increased expression of Ace2 targets such as *CHT3* and *SCW11* and, in fact, this is the case (Fig. 3b). Consistent with the increased Ace2 function in *amn1*∆∆ mutants, these strains show decreased lateral yeast formation relative to wild type cells in Spider medium at both 4 and 8 h post-hyphal induction (Fig. 3c). The hyphae of *amn1*∆∆ strains are otherwise normal true hyphae (Fig. 3d). We previously showed that *PES1* expression is increased in *ace2*∆∆ strains. In keeping with the reduced lateral yeast formation in *amn1∆∆* cells, *PES1* expression is reduced in the hyphae of *amn1*∆∆ mutants relative to wild type (Fig. 3e).

**Fig. 4:**
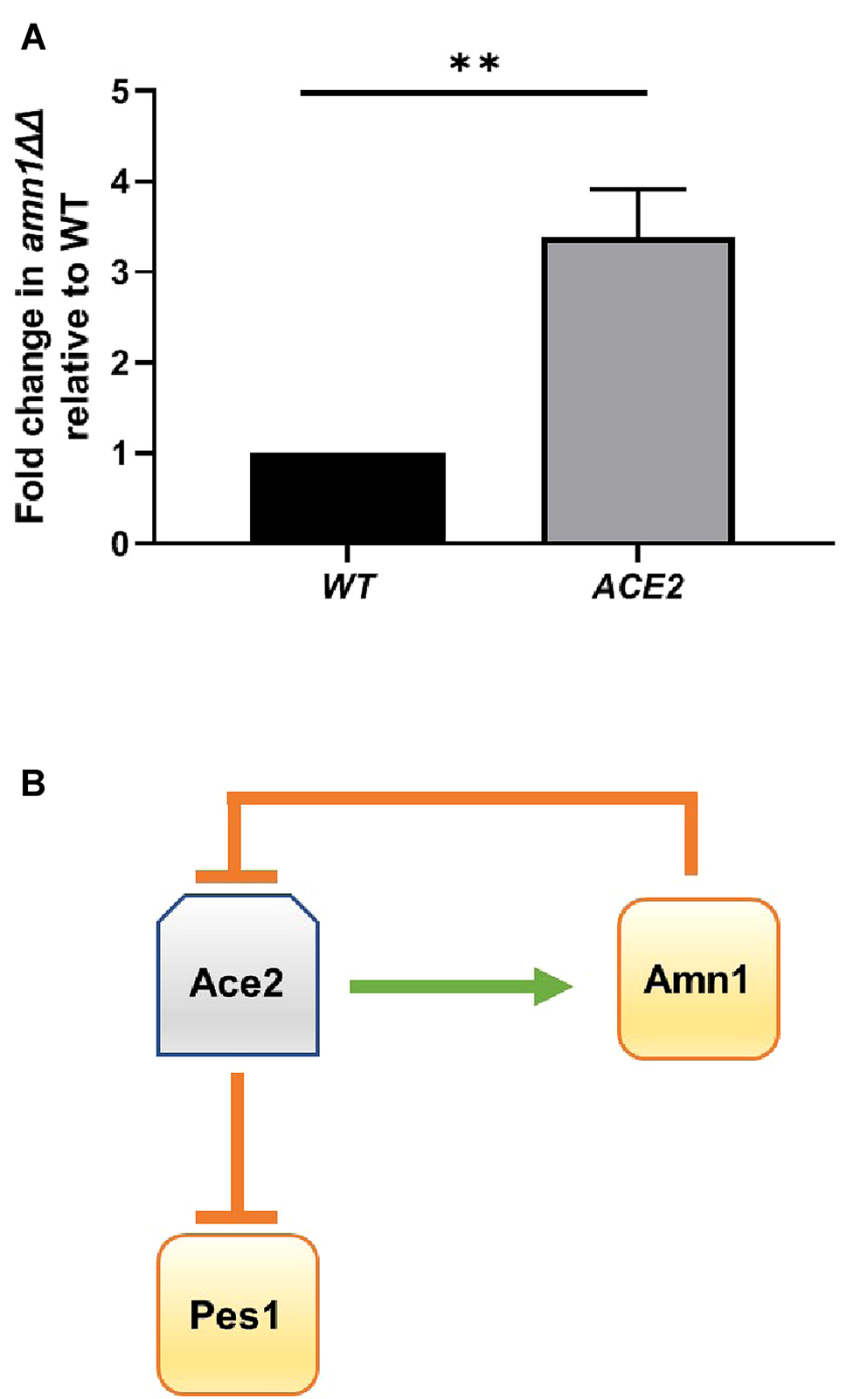
Amn1 and Ace2 form a feedback loop at the transcriptional level and regulate *PES1* expression. **a** *ACE2* expression is increased in *amn1*∆∆ mutants during hyphae induction. Bars indicate the mean ratio of the expression of the indicated gene expressed as fold-change in the ratio of mutant/wild type. Error bars indicate the standard deviation of two biological replicates performed in duplicate. Significant differences were determined by Student’s *t* test with a threshold of *P* < 0.05: **P < 0.01. **b** Data from Fig. 3a/4a and previously published data showing that Ace2 represses *PES1* expression during hyphae formation (Wakade et al. 2020) provides genetic support for the indicated feedback loop

In *S. cerevisiae*, Amn1 binds Ace2 and targets it to the E3 ligase which in turn leads to proteasomal degradation but has essentially no effect on the expression of *ACE2* RNA (Fang et al. 2018). To investigate the mechanism by which Amn1 affects Ace2 function during hyphae formation, we first compared the expression of *ACE2* in wt and *amn1*∆∆ strains after hyphal induction by qRT-PCR. Surprisingly, we found that *ACE2* expression is increased in *amn1*∆∆ by three-fold (Fig. 4a), indicating that Amn1 plays a role in the regulation of *ACE2* expression. Amn1, however, has no DNA binding sequences and, thus, its regulation of *ACE2* expression must be indirect. However, this increased expression would explain the decreased *PES1* expression in *amn1*∆∆ strains. As noted above, the effect of Amn1 on *ACE2* expression is distinct from the case in diploid *S. cerevisiae* cells under yeast phase growth. Additional experiments will be needed to dissect the relative contributions of gene expression and possible protein degradation in the role of Amn1 in *ACE2* steady state levels during hyphae formation.

Importantly, our data indicate that, unlike *S. cerevisiae* (Fang et al. 2018), there is a direct or indirect mechanism by which Amn1 inhibits the transcription of *ACE2* in *C. albicans*. It is possible that Amn1 also binds and degrades other Ace2-regulating transcription factors, such as Tec1 or Brg1. Since Ace2 induces the expression of Amn1 and Amn1 reduces the expression of Ace2, there appears to be a negative feedback loop that links Amn1 levels to *ACE2* expression (Fig. 4b). Genome-wide chromatin immunoprecipitation data have not found that Ace2 binds its own promoter (Desai et al. 2016); although this does not rule out autoregulation of *ACE2*, it suggests that other mechanisms may be operative. Ace2 does, however, bind to the promoter of *BRG1* (Desai et al. 2016) and, as we have shown, Brg1 binds to the *ACE2* promoter and is required for full expression *ACE2* expression during hyphae formation (Saputo et al. 2014; Desai et al. 2016). Thus, we suggest that Ace2 and Brg1 form a positive feedback loop that regulates the increase in *ACE2* expression after initiation of hyphal development in *C. albicans*. The steady state levels of *ACE2* expression, therefore, are determined by the relative activities of these two competing, feedback loops.

**Fig. 5:**
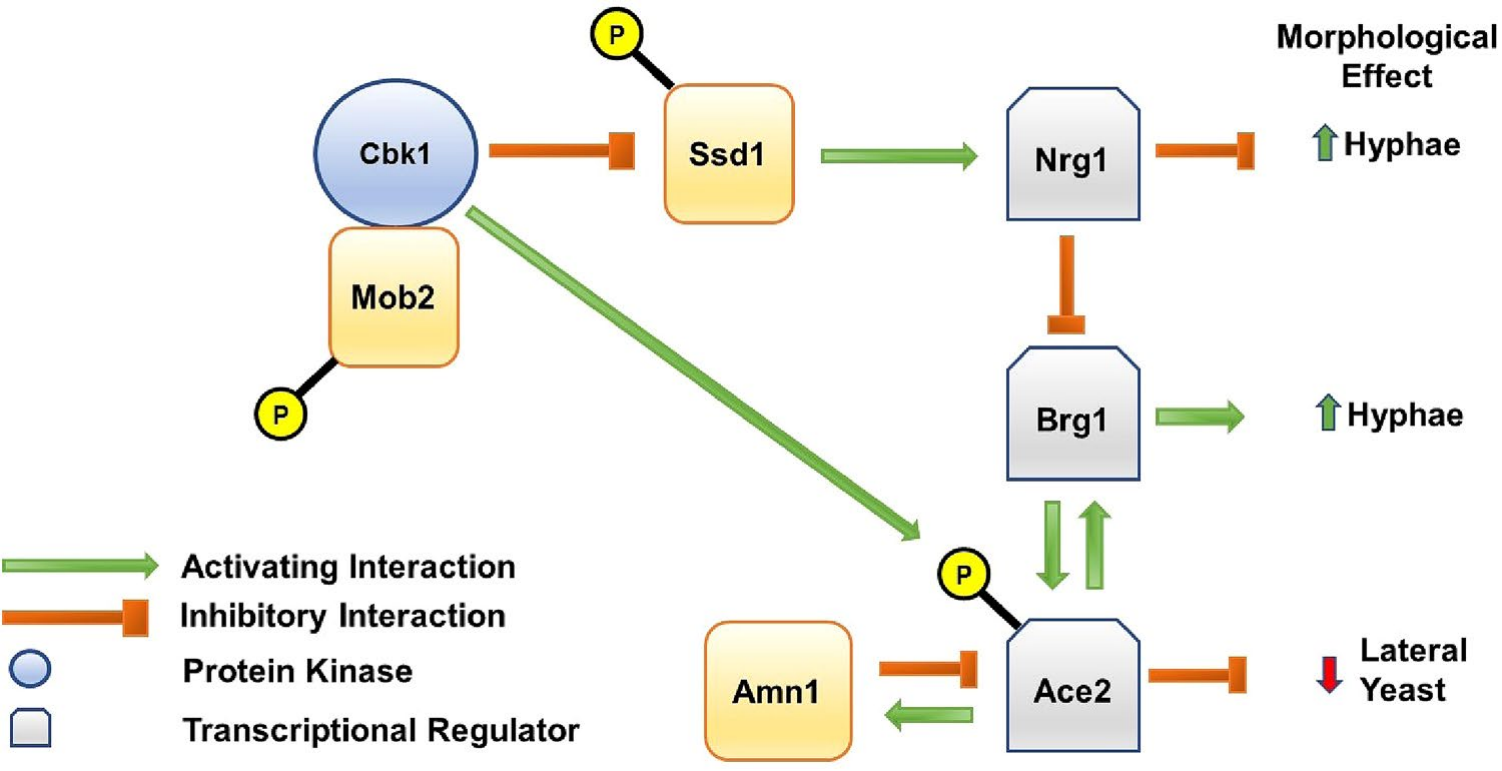
Schematic for the Cbk1-Ace2 axis during hyphal morphogenesis. Green arrows indicate activating interactions and red symbols indicate repressing interactions. The presence of a P indicates phosphorylation of the indicated protein by Cbk1

Based on these new data and previously reported results, we propose an integrated model for the regulation of *ACE2* expression (Fig. 5). During hyphal induction, the levels of the hyphal repressor Nrg1 are reduced in a Cbk1-dependent process (Lee et al. 2015). Nrg1 represses the expression of Brg1 and relief of that repression occurs early in hyphal induction (Cleary et al. 2012) leading to a dramatic increase in Brg1 expression which, along with Tec1, increases *ACE2* expression by initiating a positive feedback loop (Saputo et al. 2014; Finkel et al. 2012). This positive feedback loop appears to be balanced by a negative feedback loop through the Ace2 driven expression of *AMN1*. The Ace2-Amn1 negative feedback loop operates indirectly at the transcriptional level but may occur at the protein level as well.

Early in hyphal induction the two competing feedback loops are shifted toward a net increase in *ACE2* expression (Saputo et al. 2014). The sub-apical hyphal compartments are arrested in G1 and Ace2 expression remains high enough to suppress lateral yeast formation. After approximately 3–4 h of hyphal induction, Nrg1 expression increases and, consequently, *BRG1* expression is reduced (Cleary et al. 2012). Based on these data, we propose that the positive feedback loop maintaining *ACE2* expression is interrupted and the balance tips toward Amn1-mediated reduction in Ace2 levels. Once the negative feedback loop reduces Ace2 levels to a critical level, G1-START is initiated and lateral yeast cells then begin to emerge from the hyphae.

One of the challenges in studying these events is that hyphal development cannot be synchronized. Thus, it is important to keep in mind that the transcriptional and protein levels measured for these processes are bulk averages for cultures containing cells at varying stages of development. Time course experiments for *ACE2* gene expression show that expression peaks at 3 h and is decreasing by 5 h with changes in protein levels lagging behind gene expression (Saputo et al. 2014). Thus, within the limits of experiments using bulk cultures, the timing of increased *NRG1* expression and decreasing *ACE2* expression fits reasonably well with the beginning of lateral yeast formation. The development of single cell/hyphae reporters of the proposed events would allow high resolution testing of this model.

Additional work is required to place this model on a firm biochemical foundation but its general features are consistent with the current genetic and transcriptional data and generate hypotheses for further testing. Specifically, a number of mechanistic questions remain unanswered, including: Is Ace2 acting as a transcriptional repressor in the G1 arrested hyphal compartments? If so, does it directly regulate *PES1*? If not, then how is it repressing the emergence of lateral yeast? And finally, do similar processes occur during the emergence of dispersed yeast cells from biofilms? With respect to the latter question, *AMN1* expression is increased in mature in vivo biofilms relative to initial biofilms (Nett et al. 2009), suggesting that *AMN1* may have a role in dispersal of yeast cells from biofilms.

Our findings that two G1-related proteins, Ace2 and Amn1, affect the timing of the hyphae-to-yeast transition are consistent with previous cell biological model based on delayed G1. These data provide some of the first information regarding the molecules that are involved in regulating the G1 arrest in subapical cells of *C. albicans* hyphae. In summary, our work and that of others has clearly shown that the Cbk1-Ace2 axis plays a unique role in *C. albicans* biology in that it regulates aspects of both the initiation and termination of hyphal morphogenesis.

## Methods

### Strains, media, and cultivation

All strains are derivatives of the SN background (Noble and Johnson 2005) and were constructed by deletion of *ACE2* or *AMN1* using the transient CRISPR/Cas9 method (Min et al. 2016). Homozygous integration of the deletion cassette was confirmed by standard PCR methods as previously described (Wakade et al. 2020). Yeast peptone dextrose (YPD) and Spider medium (SM) were prepared using standard recipes (Homann et al. 2009). Strains were pre-cultured in YPD overnight at 30 °C. For yeast phase growth, the cultures were back-diluted to a cell density of 0.05–0.1 OD_600_ and incubated at 30 °C until the density had reached between 0.5 and 1 OD_600_. For hyphal induction, the overnight culture was diluted 1:50 in SM and shifted to 37 °C for induction.

### Quantitative reverse transcription-PCR (qPCR)

RNA was isolated from yeast and hyphal phase cells using RiboPure ™ kit and reverse transcribed using an iScript cDNA synthesis kit (170-8891; Bio-Rad). The qPCR reaction was performed using IQ SyberGreen supermix (170-8882; Bio-Rad). Briefly, each reaction contained 10 μ of the SYBER Green PCR master mix, 0.10 μM of the respective primers and 150 ng of cDNA as a template in a total volume of 20 μL. Data analysis was performed using 2^−ΔΔ*CT*^ method and *ACT1* was used as an internal control. Data reported here are the means from 3 independent biological replicates performed in a triplicate.

### Characterization of lateral yeast formation

SN250 or *amn1*∆∆ strains were induced to form hyphae as described above. The cells were harvested after incubation for 4 and 8 h, fixed, and then examined by light microscopy. The number of hyphae with lateral yeast cells was determined for each strain by counting at least 100 hyphae for each replicate. The data are reported as the percentage of hyphae with lateral yeast cells and are means of two independent experiments. Differences between the reference SN250 and *amn1*∆∆ were analyzed by Student’s *t* test and the *P* value was less than 0.05 indicating statistical significance.
